# Complete genome sequencing and evolutionary analyses of duck hepatitis a viruses in Egyptian duck farms

**DOI:** 10.1186/s12917-026-05607-4

**Published:** 2026-06-05

**Authors:** Nahed Yehia, Mohammed A. AbdelSabour, Dalia Said, Rania F. El Naggar, Yahia M. Madbouly, Ahmed Abd Elhalem Mohamed, Ahmed H. Salaheldin, Mohammed A. Rohaim

**Affiliations:** 1 Reference Laboratory for Veterinary Quality Control on Poultry Production, Animal Health Research Institute (AHRI), Agriculture Research Center (ARC), Giza, 12618 Egypt; 2 Department of Poultry Viral Vaccines, Veterinary Serum and Vaccine Research Institute (VSVRI), Agriculture Research Centre (ARC), Cairo, 11435 Egypt; 3 Department of Virology, Faculty of Veterinary Medicine, University of Sadat City, Sadat, 32897 Egypt; 4 Department of Poultry and Fish Diseases, Faculty of Veterinary Medicine, Alexandria University, Alexandria, 21944 Egypt; 5 Department of Virology, Faculty of Veterinary Medicine, Cairo University, Giza, 12211 Egypt

**Keywords:** DHV-1, DHV-3, Complete genome, Genetic characterisation, Vaccine update

## Abstract

**Background:**

Duck hepatitis virus (DHV) continues to pose a substantial threat to duck production worldwide, causing acute hepatitis, neurological manifestations, and elevated mortality rates, even in vaccinated flocks. During 2022-2023, a significant outbreak involving DHV-1 and DHV-3 was reported in duck farms across five governorates in North Egypt. The outbreaks primarily affected Pekin ducklings aged 4–15 days, with mortality rates ranging from 50% to 70%. Affected ducklings exhibited characteristic pathological lesions, including hepatomegaly with haemorrhages, splenomegaly, and renal enlargement.

**Results:**

A total of 30 liver and spleen samples collected from affected farms were analysed using reverse transcription PCR (RT-PCR) targeting the 3′ untranslated region (3′UTR) and the VP1 gene. DHV was detected in 56.7% (17/30) of the samples. Among the positive cases (*n* = 17), DHV-1 was identified in 29.4% (5/17), whereas DHV-3 accounted for 70.6% (12/17). Phylogenetic analysis based on whole-genome sequencing revealed that DHV-1 strains clustered within sub-clade 1a, demonstrating high genetic similarity (99.2–99.8%) with previously reported Egyptian isolates and Chinese reference strains (e.g., DHAV-1-CH-2012). In contrast, DHV-3 strains grouped within sub-clade 3a and showed close genetic relatedness to Chinese strains (97.1–98.7%), while showing marked divergence (75.9–76.3% nucleotide identity) from currently used vaccine strains. Genetic analysis identified multiple mutations in both viral types. DHV-1 exhibited amino acid substitutions within VP0, VP3, and non-structural protein regions, alongside five mutations in hypervariable region 1 (HVR1) and one mutation in HVR2 of the VP1. DHV-3 demonstrated distinct mutations within the VP1, including a unique E681K substitution identified in the Egyptian strain ND3.

**Conclusion:**

The findings demonstrate ongoing genetic evolution of DHV circulating in Egyptian duck farms, characterized by the predominance of genetically distinct DHAV-3 strains, with significant divergence from currently used vaccine strains. This genetic divergence may contribute to altered vaccine performance and continued disease outbreaks; however, its impact on vaccine-induced protection requires further experimental validation. Continuous genomic surveillance and the development of updated, strain-matched or multivalent vaccines are therefore essential to control DHV infections and minimise economic losses in Egypt’s duck industry.

## Background

Duck viral hepatitis (DVH) is an acute, highly contagious disease affecting young ducklings. The disease is caused by duck hepatitis virus (DHV; taxonomically classified as duck hepatitis A virus, DHAV), a member of the family *Picornaviridae* and genus *Avihepatovirus*. DHV is classified into three serotypes based on neutralization assays, VP1 gene divergence, and epidemiological distribution [1–3]. DHAV-1, first identified in the United States in 1949 and subsequently characterised in several countries, while DHV-2 remains largely confined to Taiwan, China. Since its first report in South Korea in 2007 and subsequent detection in China in 2008, DHV-3 has been increasingly detected, frequently co-circulating with DHV-1 [1–3]. In contrast to adult ducks, which may show mild or no symptoms, young ducklings often experience severe clinical signs, including opisthotonus, ataxia, lethargy, and sudden death. The virus spreads both vertically (from parent to offspring) and horizontally (between animals), exacerbating outbreaks in susceptible populations [4–6].

DHV is a non-enveloped virus with a positive-sense, single-stranded RNA genome of approximately 7.7 kb that encodes a large polyprotein, which is subsequently cleaved into structural and non-structural proteins. The virus comprises three major types/serotypes (DHV-1, DHV-2, and DHV-3), each exhibiting genetic variations that may influence pathogenicity and antigenic diversity [7–9]. In particular, mutations within the VP1 capsid protein, especially in hypervariable and surface-exposed regions, may alter antigenic epitopes, influencing antibody recognition and virus neutralization, which could ultimately compromise vaccine-induced protection and effectiveness [8].

Despite the widespread use of vaccines, including the attenuated E52 vaccine strain, DVH outbreaks caused by DHV-1 and DHV-3 continue to pose challenges for commercial duck production, especially in regions like Egypt, where the virus has been endemic since 1969 [10–11]. DVH outbreaks have caused significant economic losses in Egypt’s duck industry, with mortality rates reaching up to 70% in affected flocks [11]. In Egypt, DHV vaccination is commonly performed using attenuated DHAV-1-derived vaccines, including the E52 strain, administered to breeders or ducklings during the first week of life. However, vaccine efficacy remains inconsistent because of limited cross-protection against the increasingly prevalent DHV-3 type [11, 12]. In addition, co-infections with viruses such as avian influenza further complicate disease control, emphasizing the need for improved management strategies.

Recent studies suggest that current vaccination strategies may be insufficient, partly because of the incomplete understanding of DHV’s genetic diversity and evolutionary dynamics. Although previous research has focused primarily on partial sequencing of specific regions of the virus genome (e.g., 3D, VP1, and 5′ UTR), these analyses do not provide a comprehensive understanding of the virus’s evolution or its potential for genetic adaptation and antigenic variation [13]. However, the genetic diversity and evolutionary dynamics of DHV in Egypt remain poorly understood, particularly for DHV-3, thereby limiting our understanding of viral evolution and vaccine efficacy. This study aims to address this gap through whole-genome sequencing of Egyptian DHV strains (DHV-1 and DHV-3), to improve understanding of viral evolution and identify strain-specific mutations associated with virulence and vaccine efficacy. These findings may support the development of improved vaccines and more effective disease control strategies for the duck industry.

## Materials and methods

### Sample collection and processing

Between 2022 and 2023, thirty samples from clinically affected ducklings were collected during outbreaks across five governorates: Giza (8), Alexandria (4), Beni-Sueif (3), Monufia (8), and Fayoum (7) (Table 1). The investigated farms were commercial duck production systems operating under conventional management conditions. Flock sizes varied between farms, ranging from small- to medium-scale production units. All affected flocks had a documented history of routine vaccination against DHV using the attenuated E52 vaccine strain (DHV-1-derived), administered according to local veterinary practices either to breeder flocks or ducklings during the first week of life. Standard biosecurity measures, including routine cleaning, disinfection, and restricted farm access, were reportedly implemented; however, outbreaks still occurred despite these preventive measures. The number of samples per farm/region was not evenly distributed because the outbreaks varied in intensity and mortality. The sampling approach aimed to maximize representativeness of circulating strains rather than enforce equal sample numbers per region. This sampling strategy is commonly used in outbreak investigations, where sample availability is often determined by clinical presentation and mortality levels [11]. The samples were obtained from flocks exhibiting neurological signs, such as opisthotonus, ataxia, and sudden mortality (Table 1). Liver and spleen tissues were collected from each deceased duckling, pooled per individual animal, and homogenized in a saline solution containing 200 µg/ml streptomycin and 2000 IU/ml penicillin to prevent bacterial contamination. After homogenization, the samples were centrifuged at 3000 rpm for 15 min. The supernatant was stored at -80 °C for subsequent viral RNA extraction and analysis.

**Table 1:** Details of collected samples, RT-PCR results, and accession numbers of the sequenced samples

NO.	Age (day- old)	Governorate	Year	Result of RT-PCR	Accession number
1	7	Giza	2022	DHV-1	-
2	11	Alexandria	2023	DHV-3	PV014351
3	8	Monufia	2022	DHV-1	PV014354
4	4	Fayoum	2022	DHV-3	-
5	9	Monufia	2022	DHV-1	-
6	13	Monufia	2022	DHV-3	PV014352
7	6	Beni-Sueif	2023	DHV-3	-
8	9	Monufia	2022	DHV-3	-
9	5	Fayoum	2023	DHV-3	-
10	10	Beni-Sueif	2023	DHV-3	PV014353
11	8	Giza	2022	DHV-1	-
12	10	Beni-Sueif	2023	DHV-3	-
13	9	Giza	2023	DHV-3	-
14	13	Fayoum	2023	DHV-3	-
15	8	Fayoum	2023	DHV-1	-
16	5	Fayoum	2022	DHV-3	-
17	9	Fayoum	2023	DHV-3	-

### Viral RNA extraction and detection of DHV

Viral RNA was extracted from the supernatant of homogenized tissues using the QIAamp Viral RNA Mini Kit (Qiagen, GmbH, Hilden, Germany) following the manufacturer’s instructions. For DHV detection, reverse transcription PCR (RT-PCR) targeting the 3′ untranslated region (3′UTR) was performed using previously described primers [14], yielding an expected amplicon size of approximately 250 bp. To determine the DHV serotype, the VP1 gene was amplified using primers described by Liu et al. [15] and Doan et al. [16], producing amplicons of approximately 880 bp. Complementary DNA (cDNA) was synthesised from extracted RNA using a reverse transcriptase enzyme, followed by PCR amplification with Phusion High-Fidelity DNA Polymerase (Thermo Fisher Scientific, Waltham, MA, USA). Amplified products were visualized by agarose gel electrophoresis.

### Virus isolation

Due to the unavailability of specific pathogen-free (SPF) embryonated duck embryos in Egypt, commercially available embryonated duck embryos were used for virus isolation. Virus isolation was attempted for all DHV-positive samples confirmed by RT-PCR. Four representative isolates, including one DHV-1 and three DHV-3 strains, were successfully propagated in embryonated duck embryos (EDEs) aged 10–14 days and selected for further genomic characterization. For each isolate, 10 EDEs were inoculated via the allantoic cavity [9]. The propagation process was repeated over three successive passages. The eggs were candled daily for up to 7 days post-inoculation (dpi). Following incubation, allantoic fluid was collected from inoculated eggs, and RT-PCR was performed to confirm the presence of DHV. Embryos were examined for lesions associated with viral infection, including stunting, oedema, and haemorrhages, particularly in the liver, spleen, and kidneys.

### Whole-genome sequencing

Four representative DHV isolates, including one DHV-1 isolate and three DHV-3 isolates, were selected for whole-genome sequencing based on successful virus isolation and RT-PCR confirmation. Total RNA was extracted from the allantoic fluid using TRIzol™ reagent (Invitrogen, Waltham, MA) according to the manufacturer’s instructions. The RNA concentration and purity were assessed with a spectrophotometer (ND-1000, Nanodrop Technologies, Wilmington, DE), and RNA integrity was verified by electrophoresis on a 1.2% formaldehyde agarose gel stained with GelRed^®^ (Biotium, Fremont, CA). RNA samples were stored at -80 °C until further analysis.

For whole-genome sequencing, nine overlapping PCR fragments spanning the complete viral genome were amplified and sequenced using specific primers following the protocols of Shi et al. [17] and Xu et al. [18]. The expected amplicon sizes of the overlapping fragments were approximately 850 bp (F1), 920 bp (F2), 870 bp (F3), 910 bp (F4), 860 bp (F5), 940 bp (F6), 890 bp (F7), 930 bp (F8), and 780 bp (F9), ensuring whole-genome coverage with overlapping regions for accurate sequence assembly. PCR amplification was performed using Phusion High-Fidelity DNA Polymerase (Thermo Fisher Scientific) in an Applied Biosystems 2720 thermocycler (Foster City, CA) under the following cycling conditions: an initial denaturation at 98 °C for 30 s, followed by 35 cycles of denaturation at 98 °C for 10 s, annealing at 56 °C for 30 s, and extension at 72 °C for 45 s, with a final extension at 72 °C for 10 min. PCR products were purified using the Invitrogen Gel Purification Kit (Thermo Fisher Scientific, Waltham, MA) and subsequently cloned into the pMD18-T vector (TaKaRa Bio Inc., Shiga, Japan) according to standard molecular cloning procedures. Recombinant plasmids were transformed into *Escherichia coli* DH5α competent cells (Invitrogen, Waltham, MA). Positive colonies were screened by PCR, and confirmed inserts were sequenced using an Applied Biosystems 3500xl Genetic Analyzer (Thermo Fisher Scientific, Waltham, MA). The resulting sequences were assembled using SeqMan (DNASTAR), and consensus sequences were aligned with reference genomes.

### Genetic and phylogenetic analyses

Complete DHV genome sequences were aligned with reference strains from the NCBI GenBank database using BioEdit software (version 7.2) with the ClustalW alignment algorithm [19]. Phylogenetic trees were constructed using the maximum likelihood method in MEGA software (version 6.0) with 1000 bootstrap replicates [20]. Pairwise nucleotide sequence similarity was calculated using DNASTAR software (version 20).

### Mutations mapping at VP1 proteins and functional regions

The VP1 protein was specifically analysed because it is the most variable structural protein, contains key neutralizing epitopes, and is widely used as a molecular marker for classification and vaccine evaluation [11]. The VP1 nucleotide sequences were translated into their corresponding amino acid sequences using the MEGA X software (version 10.1.8) to facilitate amino acid-level comparison among strains. To visualize sequence variation patterns in the hypervariable regions (HVRs), sequence logos were generated using WebLogo (http://weblogo.threeplusone.com, *accessed on 17 August 2025)* to illustrate VP1 protein sequence conservation and variability. This approach enabled rapid identification of key amino acid differences across aligned sequences.

### Evolution and recombination analyses

Potential synonymous (dS) and non-synonymous (dN) substitutions across the VP1 gene, as well as substitutions at each codon position, were analysed to estimate the dN/dS ratio. The dN/dS ratio was derived by comparing the observed non-synonymous substitutions to synonymous ones and then adjusted for multiple substitutions using the Jukes-Cantor correction [21]. Furthermore, the Recombination Detection Program 4 (RDP4; version 4.97) software was used to identify possible recombination events [22] in the complete DHV genomes [11]. This analysis used multiple detection methods, including RDP, Geneconv, Bootscan, Maxchi, Chimaera, Siscan, and 3Seq, with a modified significance threshold of *p* < 0.05. Recombination events were considered reliable only if supported by at least five independent detection methods. For gene-specific analysis, the Synonymous-Non-Synonymous Analysis Program (SNAP) was used to estimate the dN/dS ratio for the VP1 gene.

## Results

### Clinical signs, detection of DHV and virus isolation

**Fig. 1:**
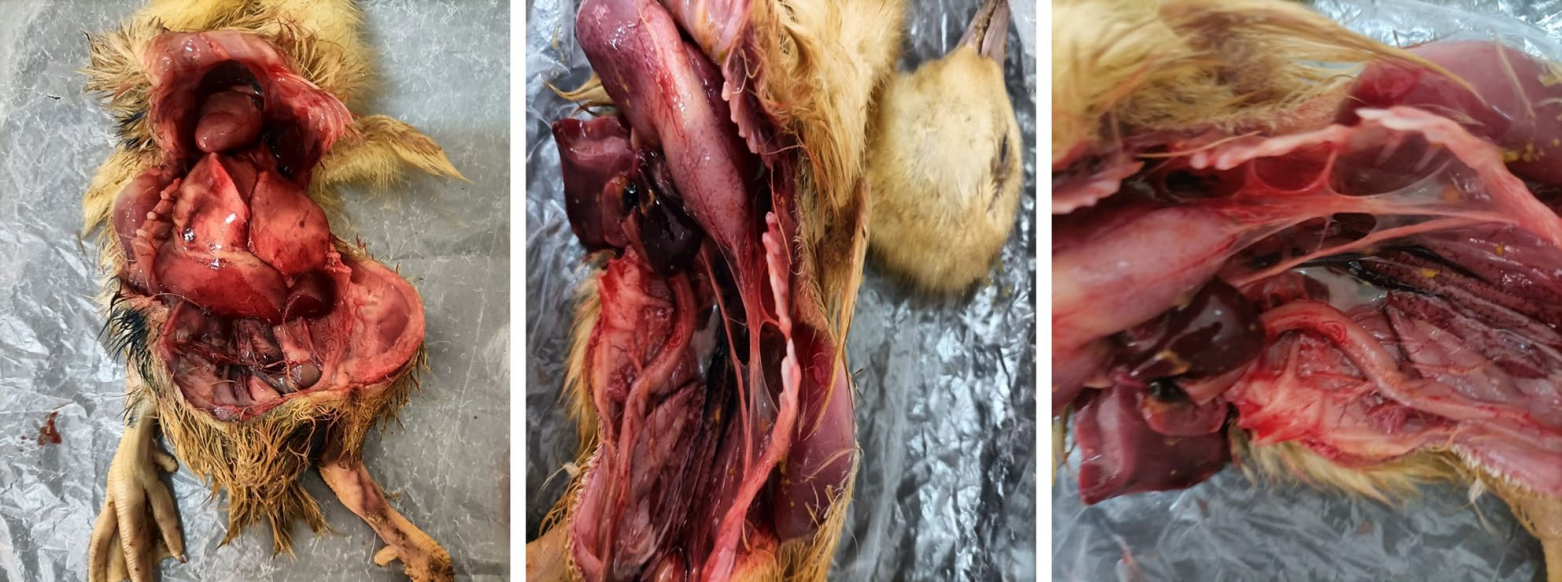
Gross postmortem lesions in ducklings infected with Duck Hepatitis Virus 1 (DHV-1) and Duck Hepatitis Virus 3 (DHV-3). The affected ducklings exhibited severe hepatomegaly with petechial and ecchymotic haemorrhages on the liver surface. The liver appeared swollen, friable, and mottled with necrotic foci, particularly in DHV-3-infected ducklings, which showed more severe hepatic damage compared to DHV-1-infected ducklings. Additionally, splenomegaly and congestion in other visceral organs, such as the kidneys and intestines, were observed, suggesting systemic viral dissemination. These findings highlight the pathogenic potential of DHV-1 and DHV-3 in infected ducklings and reinforce their role in causing acute and often fatal hepatitis

During the 2022–2023 outbreak in Egyptian duck farms, young ducklings exhibited severe clinical signs consistent with Duck Hepatitis Virus (DHV) infection (Fig. 1). Affected ducklings displayed neurological signs, including lethargy, loss of balance, opisthotonus (a condition where the head is bent backward due to muscle spasms), and ataxia (lack of muscle coordination). These signs were accompanied by high mortality rates, ranging from 50% to 70%, indicating the acute nature of the infection. Postmortem examination of ducklings infected with DHV-1 and DHV-3 revealed characteristic pathological lesions, primarily affecting the liver, spleen, and other visceral organs. The most prominent lesion observed in both infections was hepatomegaly with prominent hemorrhagic foci and necrosis. The liver appeared swollen and friable, with petechial and ecchymotic haemorrhages distributed across its surface. However, the severity of these lesions varied between the two viral types. Ducklings infected with DHV-3 exhibited more extensive hepatic necrosis and parenchymal damage than those infected with DHV-1, suggesting possible differences in virulence or pathogenic mechanism. In addition to hepatic lesion, splenomegaly was frequently observed, along with congestion in the kidneys and intestines, suggesting systemic infection.

To screen the epidemiology of DHV, 30 samples were collected from affected farms across five governorates (Giza, Alexandria, Beni-Sueif, Monufia, and Fayoum) and tested using reverse transcription PCR (RT-PCR) targeting the 3’ untranslated region (UTR) of the virus. Of these, 17 samples (56.7%) tested positive for DHV. Further typing revealed that 5 samples (29.4%) were positive for DHV-1, while 12 samples (70.6%) were positive for DHV-3, indicating a higher detection rate of DHV-3 . Geographically, DHV-1 was detected in Giza, Monufia, and Fayoum, while DHV-3 among the investigated outbreaks in Giza, Alexandria, Beni-Sueif, Monufia, and Fayoum.

To further characterize the detected viruses, RT-PCR-positive samples were used for virus isolation in embryonated duck eggs (EDEs) aged 10–14 days. The embryos were inoculated via the allantoic cavity, and propagation was repeated over three successive passages. Infected embryos exhibited signs of viral infection, including stunted growth, hepatic haemorrhages, and oedema. Between 35% and 50% of the embryos inoculated with either DHV-1 or DHV-3 died within five days post-inoculation, highlighting the high pathogenicity of both strains. By the second passage, approximately 20% of the embryos remained alive but showed organ enlargement and hepatic necrosis, consistent with the pathological effects of DHV infection. Allantoic fluid collected from infected embryos was tested using RT-PCR, confirming the presence of DHV. This successful isolation of the virus in embryonated eggs provides a reliable method for further studies on viral replication, pathogenicity, and vaccine development [9, 23–25].

### Genomic and phylogenetic analyses

Whole-genome sequencing was conducted to determine the genetic relationship between Egyptian DHV strains and global reference isolates. Phylogenetic analysis classified the Egyptian strains into two main clades: DHV-1 and DHV-3. In this study, the DHV-1-Egypt-ND4 strain belongs to sub-clade 1a and shared high nucleotide identity (99.2–99.8%) with previously reported Egyptian strains and Chinese reference strains, including DHV-1-CH-2012, DHV-1-HDHV1-ZJ, and DHV-1-HDHV1-JX, indicating limited genetic divergence. Additionally, three DHV-3 strains: DHV-3-Egypt-ND1, DHV-3-Egypt-ND2, and DHV-3-Egypt-ND3 clustered with Chinese strains DHV-3-CH-P90 and DHV-3-SD70, sharing 97.1–98.7% nucleotide identity.

The complete genomes of DHV-1 and DHV-3 were approximately 7.7-7.8 kb in length and contained a poly(A) tail at the 3' end. Both genomes contained a single large open reading frame (ORF) encoding structural proteins (VP0, VP3, VP1) and non-structural proteins (2A1–2 C, 3 A–3D). Comparative analysis showed that the VP1 gene of DHV-1-Egypt-ND4 was slightly shorter than that of DHV-3, measuring 714 nucleotides versus 720 nucleotides. Additional genomic features, including UTR lengths and polyprotein cleavage sites, are listed in Table 2.

**Table 2:** The clustering of gene sequences, a comparison of genomic characteristics, and the identification of amino acid residues at predicted C-terminal cleavage sites for DHV-1 and DHV-3 in this study

Code/region	Length		Predicted C-terminal Cleavage Sites (Amino Acid)	
	DHV-1	DHV-3	DHV-1	DHV-3
5’UTR	626	652	-	-
ORF	6750	6756	-	-
3’UTR	315	366	-	-
Poly A	18	18	-	-
Complete size	7709	7792	-	-
Poly protein	2249	2251	-	-
L	30	30	30 (L/G)	30 (L/G)
VP0	266	266	256–257 (Q/G)	256–257 (Q/G)
VP3	237	237	Q/G (493–494)	Q/G (493–494)
VP1	238	240	E/S (731–732)	E/S (733–734)
P1	701	703	E/S	E/S
2A1	20	20	NPG/P (749–752)	NPG/P (753–754)
2A2	285	285	Q/S (1036–1037)	Q/S (1038–1039)
2B	119	119	Q/S (1155–1156)	Q/S (1157–1158)
2 C	333	333	Q/S (1488–1489)	Q/G (1490–1491)
P2	757	757	Q/S	Q/G
3 A	93	93	Q/S (1581–1582)	Q/S (1583–1584)
3B	34	34	Q/S (1615–1616)	Q/G (1617–1618)
3 C	181	181	Q/G (1796–1797)	Q/G (1798–1799)
3D	453	453	-	-s
P3	761	761	-	-

**Fig. 2:**
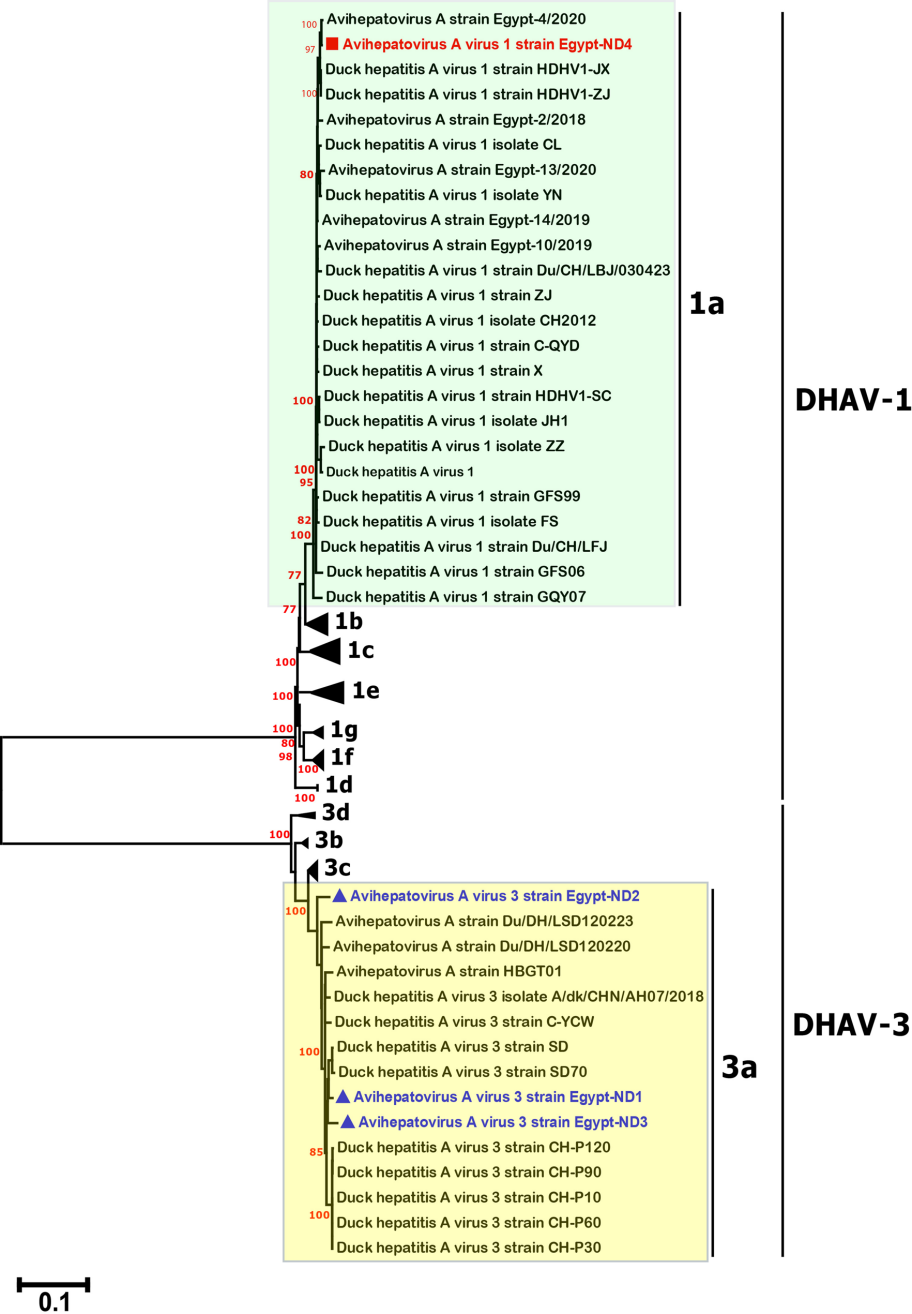
Maximum Likelihood phylogenetic tree of Duck Hepatitis Virus 1 (DHAV-1) and Duck Hepatitis Virus 3 (DHAV-3) based on whole-genome sequences. The tree was constructed using the Maximum Likelihood method implemented in MEGA 6.0 under the General Time Reversible model with gamma distribution and invariant sites (GTR + G+I), with 1,000 bootstrap replicates. Egyptian DHAV-1 and DHAV-3 isolates are highlighted in bold. Branch lengths represent the number of nucleotide substitutions per site, and bootstrap values > 70% are shown at the nodes. The tree includes representative reference sequences from the DHV-1 and DHV-3 sub-clades incorporated into the present phylogenetic dataset. Egyptian DHV isolates clustered within DHV-1 sub-clade 1a and DHV-3 sub-clade 3a

The phylogenetic tree showed that the analysed DHV strains clustered into two main groups, DHV-1 and DHV-3, based on whole-genome sequences (Fig. 2) and revealed distinct clustering patterns among Egyptian DHV-1 and DHV-3 isolates, reflecting their evolutionary relationships with global strains. DHV-1 has been classified into seven recognized sub-clades (1a–1 g), whereas DHV-3 has previously been reported to comprise five sub-clades (3a–3e). However, only four DHV-3 sub-clades were represented among the reference sequences incorporated into the present phylogenetic analysis (Fig. 2). The Egyptian DHAV-1 isolate formed a monophyletic cluster closely related to recently reported Egyptian isolates and Chinese strains (DHV-1-CH-2012 and DHV-1-HDHV1-ZJ) (Fig. 2), suggesting a recent common ancestor and limited genetic divergence. In contrast, Egyptian DHV-3 isolates grouped with previously reported Asian strains, whereas others formed a distinct lineage within the phylogenetic tree. Bootstrap values provided strong support for the major clades, confirming the robustness of the phylogenetic relationships. Measurable genetic distance was observed between Egyptian DHV-3 isolates and reference strains.

### Amino acids mutation analysis

Detailed mutation analysis was conducted to identify amino acid substitutions in the Egyptian DHV strains compared to the vaccine and reference strains (Table 2). In DHV-1-Egypt-ND4, several notable mutations were identified, including K34E in VP0, V347T, F397Y in VP3, and L562F in VP1 (Table 2). These mutations, particularly in the VP1 protein, which is critical for receptor binding and immune evasion, may contribute to the virus’s ability to evade host immune responses and persist in vaccinated populations [26, 27]. Notably, the E681K mutation within the polyprotein of the Egyptian strain ND3 was identified as a unique mutation in the hypervariable region (HVR), suggesting a possible effect on antigenic characteristics of the virus, potentially affecting vaccine-induced immune recognition [8, 11, 28]. Additionally, the QSD (Gln-Ser-Asp) motif, present in the HVR of all DHV-3 strains, may play a role in viral attachment and entry into host cells [29, 30].

**Fig. 3:**
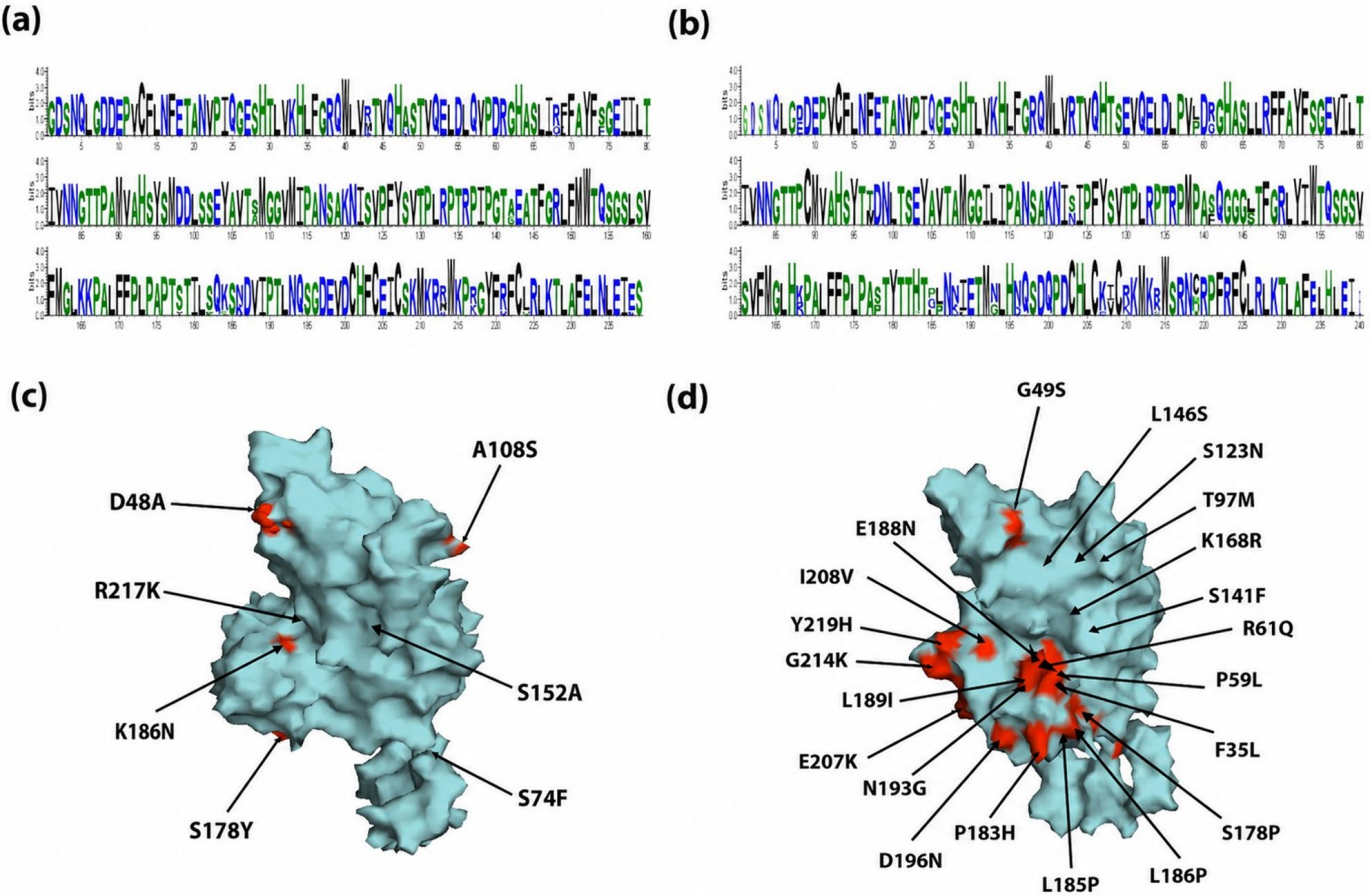
Figure 3. Sequence logo and 3D structural mapping of the VP1 protein in DHV-1 and DHV-3 isolates from Egypt. (A, B) WebLogo analysis illustrating the degree of sequence conservation within the VP1 protein of Egyptian DHV isolates. Panel (A) represents DHV-1 VP1, while panel (B) represents DHV-3 VP1. The sequence logos highlight highly conserved amino acid residues, which are likely essential for maintaining structural integrity, viral replication, and receptor binding functions. In contrast, regions exhibiting greater amino acid variability may reflect adaptive evolution under host immune pressure, particularly within putative antigenic sites. (C, D) Three-dimensional (3D) structural mapping of VP1 showing the spatial localisation of amino acid substitutions on the protein surface. Panel (C) corresponds to DHV-1 VP1 and identifies substitutions including D48A, S74F, A108S, S152A, S178Y, K186N, and R217K, highlighted in red. Panel (D) corresponds to DHV-3 VP1 and depicts substitutions including F35L, G49S, P59L, R61Q, T97M, S123N, S141F, L146S, K168R, S178P, P183H, L185P, L186P, E188N, L189I, N193G, D196N, E207K, G214K, Y219H, and I208V, highlighted in red. Most identified variable residues were surface-exposed, suggesting a possible role in antigenic variation and viral adaptation

To understand the evolutionary patterns of the VP1 protein in Duck Hepatitis Virus 1 (DHV-1) and Duck Hepatitis Virus 3 (DHV-3), we analysed sequence conservation using sequence logos. These visual representations highlight the relative conservation of amino acids at each position, providing insights into potentially important structural or functional residues. Overall, both DHV-1 (Fig. 3a) and DHV-3 (Fig. 3b) VP1 proteins showed regions of strong conservation, particularly in residues likely involved in viral infectivity, structural integrity, or host receptor interactions. However, differences between the two viral types suggest possible adaptation mechanisms, potentially driven by immune selection pressure or host-specific factors. Variability in surface-exposed regions is especially relevant, as these sites often mediate immune recognition and viral neutralization.

To further assess sequence variability, we mapped specific amino acid substitutions onto a three-dimensional surface model of DHV-1 VP1 (Fig. 3c). The identified substitutions include D48A, A108S, S152A, S74F, S178Y, K186N, and R217K, which were predominantly located on the protein surface. Several of these substitutions fall within regions that could influence antigenicity or receptor binding. For example, D48A and S74F were located near potential interaction interfaces, which might alter host receptor affinity or immune recognition, whereas S178Y and K186N are positioned within surface-exposed loops. These changes could affect viral stability or immune evasion. The presence of R217K near the C-terminal region may also have functional implications, possibly impacting VP1’s role in viral assembly or host adaptation.

A comparison with DHV-3 VP1 revealed a higher number of amino acid substitutions, suggesting more extensive antigenic evolution (Fig. 3d). Notable mutations include G49S, L146S, S123N, T97M, K168R, S141F, R61Q, P59L, F35L, S178P, L185P, L186P, P183H, D196N, N193G, E207K, L189I, G214K, Y219H, I208V, and E188N. Many of these mutations were located in surface-exposed regions, which could significantly impact immune recognition. Substitutions such as G49S, P59L, and R61Q were located within flexible loops, regions often associated with antigenic variation. Mutations such as L185P, L186P, and P183H occurred within structurally important domains, potentially affecting VP1’s folding and stability. Additionally, E188N, G214K, and Y219H are positioned in areas that might modulate host-virus interactions, enhancing viral adaptability. The increased number of substitutions in DHV-3 compared to DHV-1 suggests greater selective pressure, possibly due to host immune responses or environmental factors.

The structural and sequence analyses highlight key differences between DHV-1 and DHV-3 VP1 evolution. Although both viral types share conserved core motifs necessary for viral function, DHV-3 showed greater sequence variability, particularly in regions predicted to be antigenically relevant. This suggests that DHV-3 may be undergoing more rapid antigenic drift, potentially influencing host susceptibility and vaccine efficacy. The distribution of mutations further supports the hypothesis that immune-driven selection plays a significant role in VP1 evolution. Many mutations in DHV-3 occur at positions known to be critical for immune escape in related picornaviruses, indicating an adaptive response to host defences. This antigenic variability could have important implications for cross-protective immunity and the design of broadly effective vaccines.

### Selective pressure and recombination analyses

To investigate potential recombination events in Egyptian DHV-1 and DHV-3 strains, we employed the Recombination Detection Program 4 (RDP v4.97) using multiple detection methods, including RDP, Geneconv, Bootscan, Maxchi, Chimaera, Siscan, and 3Seq. A modified significance threshold of *p* < 0.05 was applied, and recombination events were considered reliable only if supported by at least five independent detection methods. Our analysis revealed no evidence of recombination in the whole-genome sequences of the Egyptian DHV-1 and DHV-3 strains. This suggests that the observed genetic diversity observed in these strains is more likely associated with point mutations and selection pressures rather than recombination. The absence of recombination is consistent with previous studies on DHV, which have also reported limited evidence of recombination events in this virus [14, 31]. The lack of recombination in the Egyptian strains may reflect the relatively stable genetic structure of DHV in this region, possibly due to the absence of co-circulating divergent strains that could serve as recombination partners. However, continuous surveillance is essential to monitor for potential recombination events, as the introduction of divergent strains or co-infections with other viruses could increase the likelihood of recombination in the future. 

**Fig. 4:**
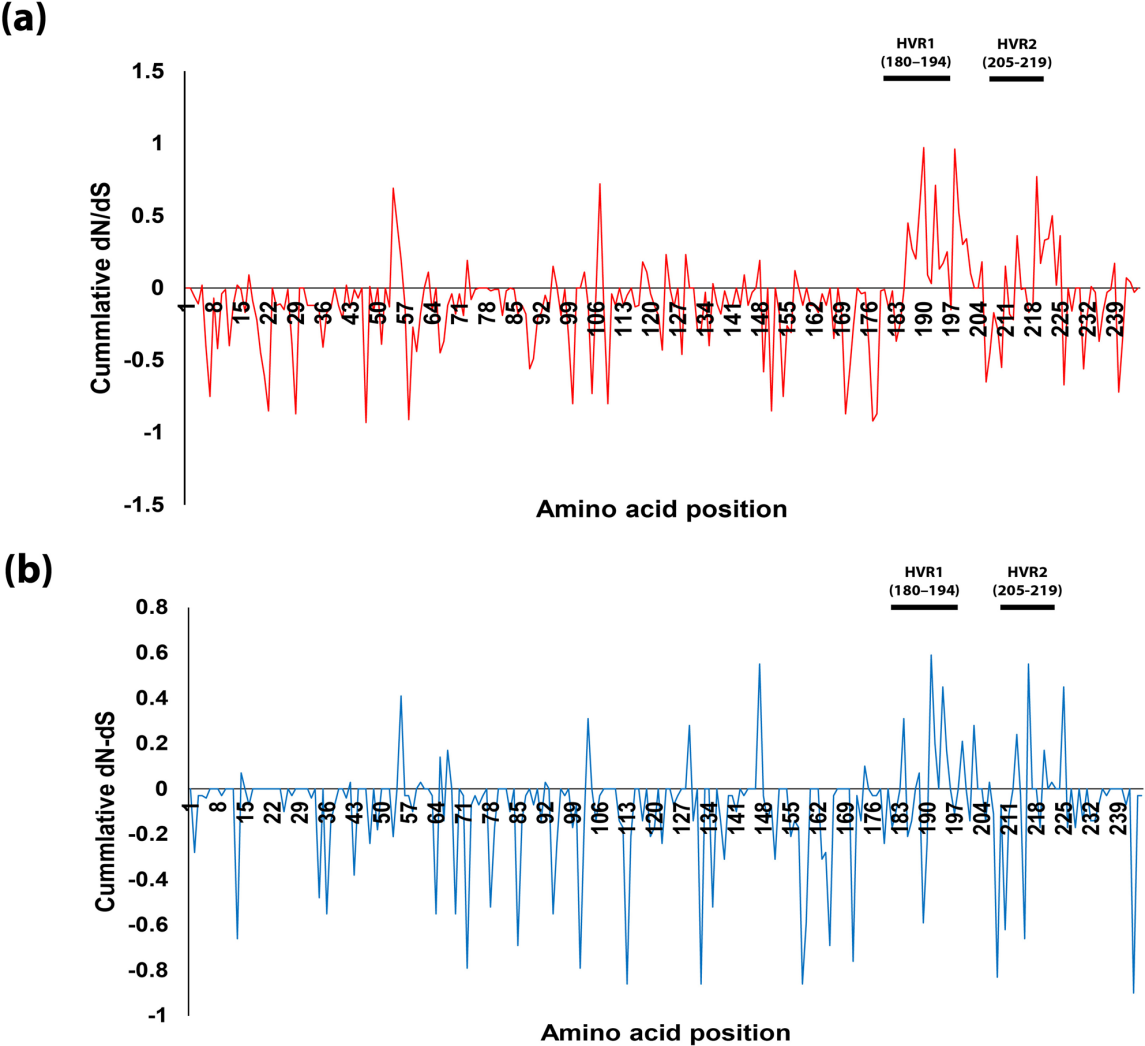
Selective pressure analysis of the VP1 gene in Duck Hepatitis Virus 1 (DHV-1) and Duck Hepatitis Virus 3 (DHV-3) isolates from Egypt. **a** Cumulative dN/dS ratio across the VP1 in DHV-1 isolates, showing variable selective pressure across amino acid positions. Two hypervariable regions (HVR1: 180–194 and HVR2: 205–219) showed higher dN/dS ratios, suggesting positive selection. **b** Cumulative dN/dS ratio across the VP1 in DHV-3 isolates, revealing a more constrained evolution with lower dN/dS ratios, indicating purifying selection across most of the protein, except for localized peaks in the hypervariable regions. These findings suggest differing evolutionary pressures between DHV-1 and DHV-3, with DHV-1 showing stronger signs of adaptive evolution, likely driven by immune selection

Selective pressure analysis of the VP1 gene in DHV-1 and DHV-3 isolates revealed distinct evolutionary patterns. The dN/dS analysis, which quantifies the ratio of non-synonymous (dN) to synonymous (dS) substitutions, showed that most regions of the VP1 were under purifying selection, as indicated by dN/dS values below 1. However, hypervariable regions (HVR1: 180–194 and HVR2: 205–219) showed elevated dN/dS ratios, suggesting localised selection . In DHV-1 isolates (Fig. 4a), positive selection signals were concentrated in these hypervariable regions suggesting adaptive evolution, potentially driven by host immune pressure. In contrast, DHV-3 isolates (Fig. 4b) displayed a more conserved evolutionary pattern, with lower dN/dS values across most of the VP1, with lower dN/dS values overall and localised peaks in the hypervariable regions. These findings suggest that VP1 evolution in DHAV-1 and DHAV-3 may be shaped by different selective pressures, although additional sequences and functional studies are needed to confirm these patterns. The difference in selection intensity between the two viruses may reflect differences in host interactions, immune evasion strategies, or evolutionary histories.

## Discussion

Duck Hepatitis Virus (DHV) continues to pose a significant threat to duck farming, particularly in Egypt, where the virus has remained endemic since 1969 [10, 11]. The present study provides important insights into the molecular epidemiology and evolutionary dynamics of DHV-1 and DHV-3 circulating in Egyptian duck farms. Among the DHV-positive samples, DHV-3 predominated over DHV-1, suggesting an increasing epidemiological importance of this genotype during recent outbreaks. Moreover, the broader geographic distribution of DHV-3 across the investigated governorates may indicate enhanced transmission dynamics, adaptation to local production systems, or greater capacity to persist under field conditions compared with DHV-1 [28, 29, 32–34]. Clinically affected ducklings exhibited severe neurological manifestations, including lethargy, loss of balance, opisthotonus, and ataxia, accompanied by mortality rates ranging from 50% to 70%. Necropsy findings, including hepatosplenomegaly and renal enlargement, were consistent with the pathological manifestations previously associated with acute DHV infection in young ducklings [1, 2].

Phylogenetic analysis demonstrated that Egyptian DHV-1 strains clustered within sub-clade 1a and remained genetically closely related to previously reported Egyptian and Chinese strains, indicating relative genomic stability and limited divergence from global isolates [28, 35]. In contrast, Egyptian DHV-3 strains clustered within sub-clade 3a and exhibited lower nucleotide identity to currently used vaccine strains than to Chinese reference strains [11, 35]. This divergence may suggest the potential for altered vaccine performance against circulating DHV-3 strains; however, the impact of these genetic differences on vaccine-induced protection requires experimental validation through challenge and neutralization studies. The close phylogenetic relationship between Egyptian and Chinese strains raises the possibility of transboundary dissemination through the movement of infected birds or contaminated materials [25, 36]. Alternatively, similar phylogenetic patterns may reflect convergent evolutionary pressures, including host immune responses and environmental conditions shaping viral adaptation across geographically distinct regions [36, 37]. Moreover, the presence of distinct phylogenetic lineages among Egyptian DHV-3 isolates may indicate localized evolutionary trajectories associated with host adaptation or production-related selective pressures [36, 37]. Although recombination was not detected in the present study, continued genomic surveillance remains essential to better understand the evolutionary mechanisms shaping DHV diversity and transmission dynamics in the field.

The amino acid substitutions identified in both DHV-1 and DHV-3 strains may have important implications for viral fitness, antigenicity, and host adaptation. In DHV-1, mutations detected in VP0, VP3, and non-structural protein regions may influence viral replication efficiency and immune evasion mechanisms [29, 38]. Notably, substitutions identified within hypervariable regions 1 (HVR1) and 2 (HVR2) of the VP1 protein are of particular interest because VP1 represents a major antigenic region involved in receptor binding and immune recognition [39–41]. These substitutions may contribute to viral persistence under immune pressure and potentially explain the continued occurrence of DVH outbreaks despite vaccination [11, 42, 43]. In DHV-3, the unique E681K substitution identified in the Egyptian strain ND3 may influence antigenic characteristics because of its location within a hypervariable region, potentially affecting vaccine-induced immune recognition [13, 43]. Furthermore, the conserved QSD (Gln-Ser-Asp) motif present in the hypervariable region of DHV-3 strains may play a functional role in viral attachment and host cell entry [18, 29, 44]. Although these findings suggest possible mechanisms contributing to immune evasion and pathogenicity, functional studies are required to confirm their biological significance and effects on viral fitness [18, 29, 44].

The genetic divergence observed between Egyptian DHV-3 strains and currently used vaccine strains may influence vaccine performance; however, experimental challenge and neutralization studies are required to determine its biological significance. The attenuated E52 vaccine strain, widely used in Egypt, is DHAV-1-derived and may provide limited cross-protection against genetically divergent DHV-3 strains [11, 12]. Although the predominance of DHV-3 during recent outbreaks could suggest reduced vaccine compatibility, the impact of genetic divergence on vaccine-induced protection remains to be experimentally validated. Nevertheless, these findings highlight the importance of considering DHV genetic diversity in future vaccine development strategies, particularly with the increasing circulation of DHV-3 strains. The development of multivalent vaccines targeting both DHV-1 and DHV-3, together with reverse genetics approaches for generating strain-matched live-attenuated vaccines, may improve protection against evolving viral populations [43, 45]. Continuous genomic surveillance will remain essential for monitoring emerging variants and guiding vaccine update strategies to maintain protection against circulating strains [11]. Interestingly, no mixed infections involving DHV-1 and DHV-3 were detected despite the co-circulation of both viral types in Egyptian duck farms. However, the relatively limited number of DHV-positive samples analysed (*n* = 17) may have reduced the probability of detecting co-infections. Consequently, the absence of mixed infections in the present dataset should not be interpreted as evidence of their absence in the field, particularly where low-abundance viral populations may escape detection using conventional molecular methods. Further investigations employing larger sample sizes and high-resolution sequencing approaches, including deep sequencing, are needed to better define the epidemiology and evolutionary significance of DHV co-infections in Egyptian duck populations [8, 46, 47].

Selective pressure analysis provided important insights into the evolutionary dynamics of DHV-1 and DHV-3 in Egyptian duck populations. The predominance of purifying selection across most regions of the VP1 gene highlights the functional constraints imposed on this structural protein, where excessive amino acid changes may compromise viral fitness. Nevertheless, positively selected sites identified within hypervariable regions suggest localised adaptive evolution, potentially associated with immune evasion or host adaptation. The stronger positive selection signals observed in DHV-1 compared with DHV-3 may indicate greater immune-driven diversification and antigenic variability, potentially facilitating immune escape and the emergence of new variants. In contrast, the lower dN/dS ratios observed in DHV-3 suggest stronger functional conservation and slower evolutionary dynamics, likely reflecting greater structural constraints on VP1. These findings have important implications for vaccine development and disease control, as hypervariable regions under positive selection may represent key determinants of immune recognition and potential vaccine mismatch. Continuous monitoring of these regions will be important for tracking viral evolution and improving understanding of viral pathogenesis, host–virus interactions, and future vaccine design.

While this study provides valuable insights into the genetic diversity and evolution of DHV strains in Egypt, several limitations should be considered. First, the relatively limited sample size (30 samples) and geographic coverage (five governorates) may not fully represent the diversity of circulating DHV strains across Egypt. Nevertheless, the selected governorates represent major duck-producing areas in North Egypt and were included based on outbreak occurrence and sample availability during the study period. Broader epidemiological investigations incorporating expanded geographic coverage and longitudinal sampling are therefore needed to better define the prevalence, evolution, and transmission dynamics of DHV across Egypt. Second, the study covered outbreaks during 2022–2023 only, limiting assessment of longer-term evolutionary trends. Third, functional studies were not performed, restricting interpretation of the biological significance of identified mutations in relation to viral pathogenicity, antigenicity, and immune evasion. Future in vitro and in vivo investigations are therefore required to validate the functional consequences of these genomic changes. In addition, diagnostic screening focused specifically on DHV-1 and DHV-3, while other pathogens associated with duckling mortality were not systematically investigated. Consequently, the contribution of viral or bacterial co-infections to disease severity cannot be excluded, and broader molecular surveillance approaches, including multiplex pathogen detection, would improve understanding of the infectious landscape associated with duckling mortality in Egypt. Finally, virus isolation was performed using non-SPF embryonated duck embryos because SPF duck embryos are not commercially available in Egypt. Although virus isolation was confirmed by RT-PCR and characteristic embryo lesions, the possibility of interference from endogenous infectious agents cannot be entirely excluded. Future studies employing SPF embryos and expanded experimental controls would further strengthen isolation reliability and improve understanding of DHV epidemiology and control strategies.

## Conclusions

This study demonstrates the continued genetic evolution of DHV circulating in Egyptian duck farms, with co-circulation of DHV-1 and predominance of genetically divergent DHV-3 strains. The observed divergence between circulating field strains and currently used vaccine strains, particularly DHV-3, highlights the importance of continuous genomic surveillance and supports the development of updated strain-matched or multivalent vaccines to improve disease prevention and control in Egypt.

## Data Availability

All sequences acquired in this study are available in NCBI GenBank under accession numbers: PV014351- PV014354. Other data that support the findings of this study are available from the corresponding author upon reasonable request.
